# Implementing a group singing intervention for postpartum depression within the Italian health service

**DOI:** 10.3389/fmed.2024.1461965

**Published:** 2024-10-21

**Authors:** Ilaria Lega, Ilaria Luzi, Simona Mastroeni, Claudia Ferraro, Silvia Andreozzi, Serena Donati, Pietro Grussu, Valentina Cavazzana, Patrizia Proietti, Patrizia Magliocchetti, Chiara Monaldi, Cristina Biglia, Rossana Oreggia, Catterina Seia, Calum Smith, Katey Warran, Nils Fietje, The Italy and GLOBUS Music and Motherhood Groups

**Affiliations:** ^1^Women’s, Children’s and Adolescents’ Health, National Centre for Disease Prevention and Health Promotion, Istituto Superiore di Sanità, Rome, Italy; ^2^Risk Factors Surveillance and Health Promotion Strategies, National Centre for Disease Prevention and Health Promotion, Istituto Superiore di Sanità, Rome, Italy; ^3^Azienda ULSS 6 Euganea, Este, Italy; ^4^Dandelion Associazione di promozione sociale, Maserà di Padova, Italy; ^5^ASL Roma 2, Rome, Italy; ^6^Studio Lago—Il centro della voce, Rome, Italy; ^7^ASL Città di Torino, Turin, Italy; ^8^Cultural Welfare Center, Turin, Italy; ^9^Nuffield Department of Population Health, University of Oxford, Oxford, United Kingdom; ^10^Consultant, Behavioural and Cultural Insights Unit, World Health Organization Regional Office for Europe, Copenhagen, Denmark; ^11^Social Biobehavioural Research Group, Research Department of Behavioural Science and Health, University College London, London, United Kingdom; ^12^School of Health in Social Science, University of Edinburgh, Edinburgh, United Kingdom; ^13^Behavioural and Cultural Insights Unit, World Health Organization Regional Office for Europe, Copenhagen, Denmark

**Keywords:** art and health, postpartum depression, family care centres, primary care, maternal health services, Implementation, singing, music

## Abstract

**Background:**

In the United Kingdom a singing intervention for Postpartum Depression (PPD) titled “Music and Motherhood” was found to be effective. The World Health Organization Regional Office for Europe coordinated a study to assess the feasibility of implementing and adapting the intervention in other countries. In Italy, recent studies have highlighted the need to promote the availability of effective interventions for PPD in primary care.

**Aim:**

To describe the implementation of “Music and Motherhood” within the Italian primary care services dedicated to pregnancy and postnatal care in three different geographical locations, thus providing an example of strategies for implementing an arts and health intervention in primary care that can improve health and well-being.

**Methods:**

A 10-week group singing intervention for mothers with PPD was conducted as part of a single arm feasibility study. Data were collected through one-to-one interviews, focus groups and questionnaires from the professionals involved in the implementation and selected participating mothers. A conceptual framework including acceptability, appropriateness, feasibility, fidelity, implementation process, costs and sustainability was adopted for analysis. Number of sessions attended by mothers and implementation outcome measures for acceptability, appropriateness and feasibility, each consisting of four items rated on a 5-point Likert scale were also gathered.

**Results:**

The intervention was found to be inclusive of women from different socio-cultural backgrounds and appropriate to the context. The group setting and the use of an arts-based intervention helped to de-medicalise the process of care maximising resources. Singing helped mothers to express their feelings and find strategies to improve interaction with their child. Attention to mothers’ needs and the co-presence of the professional singing leader and a health professional were among the key factors. The median number of sessions attended was nine out of 10. In terms of acceptability, almost 90% of the professionals were in complete agreement that they liked and approved the intervention.

**Conclusion:**

Our study adds to the evidence that an arts and health intervention proven effective in one culture and linguistic context can be adapted to another. Collaboration among health professionals and artists in the implementation process and adequate funding are instrumental in moving from project to programme level.

## 1 Introduction

Postpartum depression (PPD) is one of the most common complications of childbearing, affecting 13–15% of women who give birth (1, 2). Untreated PPD can have a profound and lasting negative impact on the health of women and children, and the lifetime costs associated with perinatal mental health problems are high (2, 3). Although pregnancy and the postnatal period provide opportunities for contact with health services, studies have shown that up to 50% of women with PPD remain undiagnosed or untreated (4). Screening programmes can increase early detection of PPD, but effectively addressing perinatal mental health issues requires overcoming several barriers to appropriate care. Pharmacological treatments may be more problematic to use while breastfeeding, and research shows that women’s preferences for PPD treatment include “talking therapies” with health professionals who take the time to listen, and peer support (5, 6). Psychotherapy may therefore play a significant role at this stage of life, but it may not be readily accessible (7). Additionally, stigma can hinder access to specialist mental health care for women during the perinatal period (8). These challenges highlight the need for interventions that reduce stigma, and promote both social and peer support in this population.

A descriptive overview provided a comprehensive insight into the potential of music-based interventions, approaches and practices to support perinatal mental health in a multitude of ways. These include the mitigation of anxiety and pain during labour, the alleviation of anxiety symptoms in pregnancy, the reduction of postnatal depression symptoms and the fostering of maternal–infant bonding (9). Among the included evidence, a randomized controlled trial (RCT) conducted in Germany showed promising results with regard to the impact of music and singing interventions on maternal well-being and perceived closeness during pregnancy (10). In the UK, a three-arm RCT compared the effects of group singing, group creative play or usual care on PPD symptoms in new mothers. The study found that the singing intervention led to rapidly recovery for women with moderate-to-severe PPD compared to the usual care group (11). While both the singing and play groups shared some mechanisms, the singing group had additional specific features that allowed women to feel a sense of achievement, relaxation, and immersion, providing ‘me time’ and relief from the concerns of motherhood (12). In addition, group singing was perceived as an authentic, social and multicultural experience that calmed the baby and strengthened the mother–child bond, with mothers attending more singing sessions on average (13).

The prevalence of PPD is around 10–13% in Italy (14, 15), where suicide has been identified as a major cause of maternal death within 1 year of the end of the pregnancy (16). Moreover, lack of continuity of care between maternity and mental health services has been frequently observed, even in cases of more severe mental disorders. Family Care Centres are the primary care services of the Italian National Health Service (NHS) dedicated to low-risk pregnancy and postnatal care. Widespread in the country at a rate of 1 per 32,000 inhabitants (17), Family Care Centres are based on a multidisciplinary, proactive and holistic approach, providing mothers and families with free pre- and postnatal care including maternal and foetal assessment, antenatal classes, breastfeeding promotion and postpartum support. Family Care Centres depend on the Local Health Unit, the local public body responsible for the management and provision of healthcare services within the Italian NHS. Since 2017, the Italian Ministry of Health has established that psychological assessment for the prevention and early recognition of mental disorders have to be included in the care provided by the NHS to women in the perinatal period. Furthermore, prevention, early detection and treatment of PPD provided at the primary care level are within the objectives of the Italian national policy [Investing early in health: actions and strategies in the first thousand days of life] (18). This approach aligns with the recommendations of the World Health Organization (WHO) guideline on Early Child Development (19) and of the WHO Guide for integration of perinatal mental health in maternal and child health services (20). A recent national survey coordinated by the Istituto Superiore di Sanità found that only about 60% of Family Care Centres offer on-site treatment to women with PPD symptoms (21). This highlights the need for initiatives to promote the availability of effective PPD interventions at the Family Care Centre level, using group approaches and community resources.

In this scenario, the Istituto Superiore di Sanità participated in the “Music and Motherhood” project coordinated by the WHO Regional Office for Europe (WHO Europe) (22, 23). The project aimed to assess the feasibility of implementing the 10-week group singing intervention for mothers with PPD symptoms, shown to be effective in the UK (11), in different cultural contexts (i.e., Denmark, Italy and Romania). This is particularly important in view of the argument that arts and health activities are “complex” interventions (24), whereby it is important to consider how the intervention interacts with the context in which it is delivered, including the differing behaviours of participants (25). Preliminary results showed that “Music and Motherhood” was successfully adapted to the different cultural contexts and that the perceived impact on the mental health of participating mothers was extremely positive^1^.

The present study aims to describe the implementation process of a singing intervention for women with PPD symptoms within the Italian NHS, and to inform health professionals and service providers about the implementation strategies of an arts and health intervention in primary care settings. The findings investigating the perceived impact of the intervention on all participating mothers will be published in an upcoming WHO Europe report bringing together findings across several data collection sites, including data from Romania and Denmark.

## 2 Materials and methods

We conducted a single-arm feasibility study as part of a broader mixed-methods project coordinated by WHO Europe (22). The Italian protocol was approved by the National Ethics committee at the Istituto Superiore di Sanità (CEN 0051195) and by the Ethics Review Committee at the WHO (ERC 0003875).

### 2.1 Study setting

A central implementation team was established at the Istituto Superiore di Sanità from October 2022. The Istituto Superiore di Sanità implementation team liaised with the WHO Europe project co-ordinators, prepared the tools and materials for the study and, based on a national network of Family Care Centres, identified the primary care services in which the singing intervention could be implemented. The Family Care Centres of the Local Health Units ASL Città di Torino, ULSS 6 Euganea (in Este) and ASL Roma 2 were selected for their previous successful collaboration in research projects, their ability to implement the intervention with local resources and their different geographical location and catchment area. ASL Città di Torino covers the entire territory of the city of Turin, located in north-western Italy, with a population of approximately 872,000. ULSS 6 Euganea covers 101 small municipalities in the province of Padua, in the north-east of the country, with a total population of around 930,000. ASL Roma 2 serves the south-east of Rome, an area with a population of around 1,300,000.

Mandatory use of masks as Personal Protective Equipment in healthcare facilities due to COVID-19 pandemic (26), which ran in Italy until 30^th^ June 2023, required the identification of non-medical venues in which to hold the singing groups: in Turin, a room in an historic music library in a municipal villa; in Este, a gymnasium attached to the Family Care Centre; and in Rome, a room in the administrative building of the Local Health Unit. The locations had space for 8–12 prams, adequate facilities for baby changing, easy access by public transport and/or easy parking.

### 2.2 Patient and public involvement

The project coordinators at WHO Europe and the project managers of the studies conducted in Denmark and Romania contributed to the Italian adaptation of the already developed protocol (22). The final version of the Italian protocol was draft by the Istituto Superiore di Sanità in collaboration with the health professionals from the three Local Health Units, national experts in arts and health interventions and a group of new mothers. These groups met periodically in the preparatory phase, advising on issues such as referral pathways, location, timing, and safeguarding.

### 2.3 Participants

In order to examine how the intervention was delivered, the present study included as participants both the professionals who managed and supported the implementation of the intervention at the local level (the local implementation team) and a subgroup of mothers who attended the singing sessions at the three Local Health Units and volunteered to be interviewed (the first volunteers were selected).

The local implementation team included the following professionals: the local project manager, the professionals who helped to deliver the intervention, the referrers (i.e., the midwives or other health professionals who identified the mothers for the intervention at the Family Care Centres) and the professional singing leader. The team consisted of seven professionals in Turin, four in Este and nine in Rome, for a total number of 20 professionals. As the intervention training was delivered in English, the understanding of the English language was an inclusion criterion for the professionals.

Mothers participating in the singing sessions were women in the postnatal period who were in contact with the Family Care Centres of the three Local Health Units. In accordance with the international protocol (20), the inclusion criteria were: (i) Age ≥ 18 years; (ii) EPDS score ≥ 10 at screening; (iii) to be up to 40 weeks post-birth with an infant aged 0–9 months; (iv) to be able to give informed consent in Italian. Mothers were not required to speak English because both the consent process and singing classes were conducted in Italian.

As a study focusing primarily on implementation, receiving other treatments was not an exclusion criterion. Eligible women were informed of the study by a designated health professional at the Family Care Centre; fliers describing the study were also posted in the waiting room. Based on the size of the successful singing groups in the UK, each Local Health Unit aimed to enrol the first 12 mothers who met the inclusion criteria. All women offered the singing intervention agreed to participate. Women who were willing to participate but did not meet the inclusion criteria were referred to other support activities provided by the Family Care Centres and, if necessary, to mental health assessment.

### 2.4 The singing intervention

The design and structure of the singing sessions were modelled on the arts and health intervention “Music and Motherhood” as adopted by the WHO Europe study (22, 23). Key elements included the central position of the mother as the recipient of the intervention and the experience of symptoms of PPD by all participants. Although mothers were invited to attend the sessions with their babies, this was not a prerequisite for their participation. This is because, in contrast with other mother-baby singing groups where the principal objective is for the mothers to sing for their babies, the primary objective of this intervention is the mothers themselves. The sessions, led by a professional singer leader with previous experience in the arts and health sector, promoted levelling and solidarity, included songs in different languages, physical and vocal warm ups, and harmonies and rounds, and used techniques to facilitate bonding between mother and baby. The mothers were encouraged to engage in physical contact with their babies, such as hugging or stroking, while participating in the musical activities. This included standing and moving with their babies, as well as gently bouncing them in their arms. In some instances, percussion instruments, such as maracas and drums, were incorporated into the musical performance. Additionally, other simple instruments or objects, such as scarves or wrapping paper, were utilized by the mothers and infants together (13).

In each Local Health Unit, weekly singing sessions of about 90 min were held for 10 consecutive weeks, for a total of 10 sessions. Classes included time for socialising before, during and after the singing session, facilitated by the provision of refreshments. The songs covered a range of genres and languages, occasionally incorporating instruments (e.g., guitar, ocean drum, koshi chimes…) in accordance with the preferences of the professional singing leaders and the groups at each Local Health Unit. In the Italian adaptation, at least one Family Care Centre health professional attended each meeting acting as a support figure for the professional singing leader and as a liaison between the participants and the health service. A link worker from the voluntary sector acted as liaison between the health service, the professional singing leader and the venue provider in one of the three Local Health Units.

The local and the Istituto Superiore di Sanità implementing teams received 9 h online training delivered over 2 days by Breathe Arts Health Research, an organisation with extensive experience in implementing singing for postpartum depression classes in the UK.

### 2.5 Safeguarding procedure

Prior to the start of each group, a clear and timely referral pathway was put in place to be activated in the event of any concerns for the mental health of participating mothers, or at their request, through contact with a mental health professional of the Family Care Centre or of the Local Health Unit.

### 2.6 Data collection

The qualitative component, which aimed to provide an in-depth understanding of the implementation process included at each Local Health Unit:

- Focus groups conducted with key professionals in the local implementation teams;
- One-to-one interviews with the professional singing leaders;
- One-to-one interviews with a subgroup of the mothers who attended the intervention.

The focus groups and one-to-one interviews were based on the topic guides developed by the international group (22, 23). The focus groups with the Family Care Centre professionals, lasting approximately 90 min, and the one-to-one interviews with the professional singing leaders, lasting approximately 45 min, were conducted by a researcher of the Istituto Superiore di Sanità (ILe) using Microsoft Teams®. The one-to-one interviews with the mothers were conducted by a mental health specialist from each Local Health Unit, in person or using Microsoft Teams®, and lasted approximately 30 min.

Participants read an information sheet and provided written informed consent before their participation.

A 90-min training on the qualitative procedures of the project was delivered online to the Istituto Superiore di Sanità research team (ILe, ILu, CF, and SM) by one of the international project coordinators (KW).

Quantitative data on implementation included:

- Number of singing sessions attended by the mothers and attrition (number of mothers who did not complete the singing intervention);
- Implementation short survey consisting of implementation outcome measures based on the Acceptability of Intervention Measure (AIM), the Intervention Appropriateness Measure (IAM), and the Feasibility of Intervention Measure (FIM), commonly used as early indicators of implementation success in formative research or pilot studies (27). Each measure consists of four-item rated on a 5-point Likert scale scored 1 (“strongly disagree”) to 5 (“strongly agree”) and ranges from 4 to 20, with higher scores indicating greater readiness for implementation.
  Information about the professionals (e.g., gender, age, and years of acquired experience) were also included in the questionnaire.

All data were collected by Istituto Superiore di Sanità within 5 weeks and 3 days after the last singing session, except for the attendance data that was recorded by the Support staff.

### 2.7 Data analysis

The focus groups and semi-structured one-to-one interviews were audio-recorded. Three members of the central implementation team created verbatim transcriptions and coded the transcripts (ILu, ILe, CF). Framework Method was used to analyse the transcripts (28), whereby an analytic framework provided by the international coordinating group was used to chart data into pre-existing themes that had been constructed using theories from implementation science (22, 23, 29, 30). The meaning units were selected and labelled according to the analytic framework. While this was primarily a deductive approach, the team complemented the framework with inductive coding when any new or interesting findings occurred. Once the initial coding was completed, the team discussed the assignment of the codes and the inclusion of emerging issues across the data from the Family Care Centres. The sub-themes to which the meaning units were then clustered largely coincided with the criteria of analysis in the framework shared by the international coordinating group, focusing on the features of the delivered intervention associated with implementation outcomes (22, 23, 29, 30)—hence the sub-themes were then grouped into the following six overarching themes: acceptability, appropriateness, feasibility, fidelity, implementation process, costs and sustainability (Table 1). Analysis was done using NVivo Release 1.7.1. Participating professionals were asked for feedback on the study findings.

**Table 1:** Themes and sub-themes related to the implementation of “Music and Motherhood” in Italy.

Themes	Subthemes and description
Acceptability	Reasons for taking part—why mothers decided to join the program
	Content and structure of the intervention—whether the singing programme is perceived agreeable and satisfactory by women with PPD symptoms
Appropriateness	Relevance to PPD symptoms management—the perceived fit and relevance of the group singing programme for managing PPD symptoms
	Core programme contents—reflections on specific aspects of programme content perceived as essential for supporting symptoms of PPD
Feasibility	Attendance—whether it was feasible for participants to attend the group singing programme to support PPD symptoms
Fidelity	Receipt and adaptations—extent to which programme was received as it was intended, e.g., how easy/difficult did participants find it to engage; any requested changes to the singing programme, e.g., not bringing their babies. Whether any changes have been made to programme delivery, e.g., lesson length, location, singing leader
Implementation process	Strategies adopted—methods or techniques used to enhance and promote adoption, implementation or sustainability of the programme, e.g., strategies used to identify and refer patients into the singing classes
	Training—feedback on the received training *(only for professionals)*
Costs and sustainability	Associated costs—any direct or indirect cost incurred for mothers as a result of attending, or extra resources/time required from local implementation groups
	Long-term sustainability—factors related to the sustainable delivery of the singing programme over time
	Mothers’ intention to adopt the intervention—whether mothers intend to continue aspects of the classes now that they are over
	Contextual or structural factors—factors that may have acted as facilitators or barriers to the implementation or delivery of the singing programme

Quantitative data were described as mean and standard deviation, median and Inter Quartile Range (IQR) for continuous variables (i.e., number of singing sessions attended, implementation outcome measures, age and years of acquired experience of professionals) as number and percentage for categorical variables (i.e., gender of professionals, 5-point Likert items of implementation measures). Stata Statistical Software (Release 17. College Station, TX: StataCorp LLC) was used for analysis.

## 3 Results

From the initial adaptation of the international protocol to the completion of the qualitative data collection, the study took a total of 9 months. A total of 23 mothers participated in the singing groups in Italy with their babies, six in the Este group, nine in the Turin group and eight in the Rome group. The mean age of the mothers was 36.0 years (SD = 5.2), with a mean age of their babies of 4.3 months (SD = 1.8). The majority of the women were highly educated (82.6%) and the mean EPDS score at the start of intervention was 15.0 (SD = 4.6).

**Figure 1:**
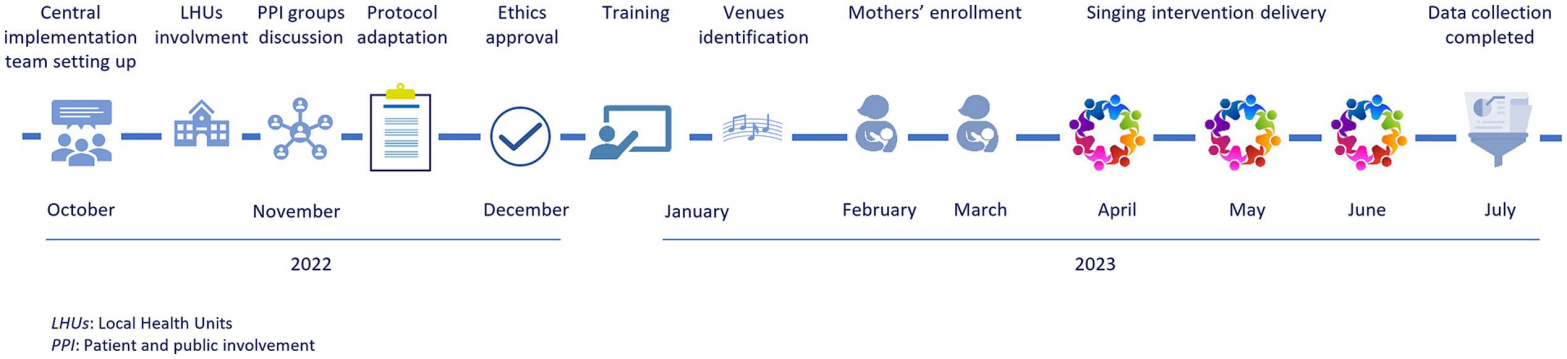
Timeframe for implementation.

The implementation times for the different phases are described in Figure 1. The national analyses were carried out by Istituto Superiore di Sanità between July and December 2023.

Table 2 presents the number of participants who took part in the data collection on the implementation process, by role and data collection method used.

**Table 2:** Role and number of participants per data collection method.

	Implementation focus group	One-to-one interview	Implementation short survey
	(*N* = 11)	(*N* = 10)	(*N* = 18)
	*N*.	*N*.	*N*.
Role			
Local project manager	3	-	3
Local support staff	5	-	6
Referrer	1	-	2
Mental health specialist	2	-	4
Professional singing leader	-	3	3
Mothers	-	7	-

It should be noted that the local project manager also served as a referrer and mental health specialist in one Local Health Unit. Among the seven mothers who volunteered, three attended the singing classes in Este, two in Turin and two in Rome. Five of them were interviewed personally and two online.

Table 3 provides a socio-demographic description of the 18 professionals from the local implementation teams who completed the short implementation survey. Of these, 14 also participated in the focus groups or one-to-one interviews.

**Table 3:** Socio-demographic characteristics of professionals.

Characteristics	All
	(*N* = 18)
Age, years	
Mean (SD)	49.8 (11.2)
Median (IQR)	55 (45–57)
Gender, %	
Females	16 (88.9)
Males	2 (11.1)
Years of acquired experience, years	
Mean (SD)	20.6 (12.0)
Median (IQR)	26 (9–31)

SD, standard deviation; IQR, interquartile range.

### 3.1 Qualitative component

The following findings relate to the six overarching themes supported by 12 sub-themes, according to the adopted analytic framework, detailed in Table 1. More examples of quotes from the focus groups and semi-structured one-to-one interviews are presented in Supplementary material.

#### 3.1.1 Acceptability

##### 3.1.1.1 Reasons for taking part

Mothers reported that they were encouraged to participate to meet other new mothers experiencing similar emotions, to share a new activity with their child, and to break the loneliness of caregiving by leaving home. The decision to participate was influenced by the trusting relationship established with the health professionals at the Family Care Centre:

*I used to go to the Family Care Centre to have check-ups for [the baby]… the midwife strongly recommended this project. Let’s say that… at the beginning I was a bit sceptical… (Mother Group 3 ID 18).*

The non-medical approach of the intervention also motivated the women to participate: “I needed to detoxify myself from the medical environment” (Mother Group 3 ID 14). Some of the mothers found the musical approach to be very appealing: “the first reason is that I really enjoy singing” (Mother Group 2 ID 12). “The free of charge nature of the intervention” (Mother Group 3 ID 14) also played an important role.

##### 3.1.1.2 Content and structure of the intervention

According to health professionals, the intervention was inclusive and suitable for women with different interests and socio-cultural characteristics, overcoming possible prejudices or difficulties such as cultural and language barriers:

*Singing and music are (…) a non-verbal instrument that really reaches in a (…) transversal, universal way (…) we are always careful, aren’t we? Obviously, for immigrant women, for foreigners, it is an absolutely very important and fundamental instrument. (Mental health professional Group 3)*

According to the professional singing leaders, the structure of the intervention met the needs of mothers with PPD symptoms by giving them the opportunity to share group activities and express their feelings:

*So it is about offering a group activity at a time when isolation is one of the worst enemies (…). One mother told me: ‘I don’t know if a discussion group would have helped me at that time, because sometimes when I’m in psychotherapy groups I feel less than others… instead having an activity to do together allowed me to be on an equal footing and then, slowly, to tell my story’ (Singing leader Group 3)*

The choice of simple songs made the sessions enjoyable and relaxing: “they did not have to, for example, think about the lyrics. Because thinking only about the melody is easier (…). The simpler the song was, the more they were able to jump in” (Singing leader Group 2).

#### 3.1.2 Appropriateness

##### 3.1.2.1 Relevance to PPD symptoms management

The implementation teams considered the intervention to be “very appropriate” (Project manager Group 2) to improve quality of life of women with PPD symptoms, offering them a new way of communicating with their child, and creating “a sense of community and identity” (Project manager Group 2). A reduction in depressive symptoms was observed “in a very short time” (Singing leader Group 2), particularly among the most socially isolated participants:

*She benefited more afterwards because she had no other connections (…). For those who really have few relationships, it is perhaps even more visible that being together in a group, it is in some way a caring factor. (Singing leader Group 3)*

Participants recommended making the intervention available to new mothers with emotional distress:

*It’s a project that should be a milestone (…) in all Local Health Units, for every mother who has a newborn. If you can participate in a project like this, it is a good thing. (Mother Group 2 ID 8)*

One mother pointed out that music was a key factor for the appropriateness of the intervention. Singing helped to express feelings without talking about them, and encouraged mothers to find strategies to improve their interaction with their baby:

*Music certainly was the key (…) if you want to, get personal, but if you don't want to, stick to the music (…) children listen to it (…) this course left me with… this motivation to find strategies, you know, in the music, in the, in … finding something that can entertain [the baby], that can calm [the baby] down. (Mother Group 3 ID 18)*

As women are experiencing depressive symptoms, it is important that the intervention takes place in close connection with a clinical network, which can ensure prompt treatment of severe depressive symptoms when needed:

*It’s like the trapeze artist, you have to put the net down and then you can do it. I mean (…) I am the first to suggest this intervention, but you have to work with the net, the net in the circus sense. (Project manager Group 1)*

##### 3.1.2.2 Core programme contents

Some elements were considered fundamental for the success of the classes, both by professionals and by mothers. Firstly, the focus on mothers and their needs. For this reason, the professional singing leader found songs that reflected the mothers’ musical tastes and origin:

*So at one point, I’m really thinking of a particular lullaby, I just took it out. I saw that when we sang it they were kind of off… and we replaced it. (Singing leader Group 3)*

*I made this song in Spanish, just for the [country of origin] girl (…) You could see right away that she was singing and that she knew it, and she was happy, you could see it in her eyes. (Singing leader Group 2)*

The attention paid by the implementation teams in making the mothers feel welcome and comfortable also contributed to the success of the intervention. Classes were held in easily accessible and well-equipped venues and mothers received refreshments and practical assistance with their child’s needs. Participants appreciated the opportunity to see their time respected, to stay and chat at the end of the session and to feel cared for:

*They would let you stay (…) there was so much care that we all appreciated so much (…) often I would stop before I left, drink the water, change [the baby] (…) a class that lasts for one hour and then you have to leave, it becomes just one more commitment among the others that you have to make (…) This instead was something where you could really take care of yourself and the baby. (Mother Group 3 ID 18)*

The co-presence of the professional singing leader and at least one health professional within the Family Care Centre made the classes a valuable opportunity to address the multiple needs of the participating mothers. While the singing leader was free to concentrate on the music, the health professionals provided practical support, answered mothers’ questions concerning the postnatal period (e.g., breastfeeding, child development) at the end of the meeting, and supported with the supervision and safeguarding of mothers experiencing symptoms of PPD. This included liaising with a mental health specialist for extra advice and support when necessary. Further, both the midwife and the social health educator were appropriate support figures:

*It did a lot to have a dedicated figure (…) having the midwife figure was also important to give those little reinforcements, because she gave all-round advice at the end. (Project manager Group 2)*

The group context, which encouraged peer support, and the collaboration of the health professional with the third sector to provide access to an artistic intervention, contributed to de-medicalisation while maximising the use of resources:

*The importance of group activities, which maximise resources. The importance of drawing on the resources of the voluntary sector (…) and then the use of de-medicalisation approaches… (Project manager Group 1)*

*The Family Care Centre could be an optimal setting for a wide adhesion, removing all the stigmatisation that exists and therefore also giving it a non-psychopathological connotation, and also mitigated by an idea of help based on singing and therefore even more non-labelling. Because it's one thing to say (…) I'm going to the singing class at the Family Care Centre—That’s nice, I’ll come too, as opposed to I have to go to the psychotherapy group (…) for “sad mothers”. (Project manager Group 1)*

#### 3.1.3 Feasibility

##### 3.1.3.1 Attendance

Overall, professionals within the Family Care Centre considered a total number of 10 weekly sessions adequate and

*Necessary to create this group cohesion and… Maybe I wouldn’t make it too long: I would give more mothers the opportunity to follow it. The result was successful, in my opinion, precisely because of these 10 sessions, which were more than enough to work well with the group. (Support staff Group 3)*

The professional singing leaders showed some perplexity about the conclusion at the end of the 10^th^ session, as if they had “abandoned” the mothers. One of them suggested adding an extra follow-up session (“I think it could be useful, at least to have a session after a while, to say *how are you?*”) and scheduling the intervention to end before the summer or Christmas holidays, for example, so that it would be easier to get into the state of mind of having reached the end of a path: “ending just before the summer organised a bit the fact that we were not really going to continue anyway (…) Because I think they would have continued anyway” (Singing leader Group 3).

Weekly class attendance was considered to be appropriate by all the actors involved: “they participated continuously as we requested” (Support staff Group 3) and absences were generally due to health reasons: “for the vaccination or when the baby (…) was sick” (Support staff Group 2). The extension of the sessions’ length if necessary facilitated the participation:

*The duration was right. Also because we were very flexible with the timetable, because it is not easy to be on time with small children. (Mother Group 2 ID 12)*

#### 3.1.4 Fidelity

##### 3.1.4.1 Receipt and adaptations

The dialogical approach of the professional singing leaders and the presence of local support staff allowed for responsive solutions to the groups’ needs. Flexibility in timing was required: the classes usually started a little later than the scheduled time in order to create a relaxed environment, and at the end of the sessions time was left for the mothers to talk to each other, attend to the child’s needs, or interact with the staff:

*We let the whole thing end smoothly and spontaneously (…) we let them leave the venue and the experience in their own time. (Project manager Group 1)*

The presence of an health professional allowed the intervention to run smoothly and reassured the participants:

*It is a great advantage to have several operators to support the singing leader, so that the mothers know that if the child cries, if sleeps, if wants something, they do not have to feel guilty that [the child] is disturbing (…) there are professionals who can take care of the children properly while they can continue to sing. (Project manager Group 1)*

While respecting the planned structure, the professional singing leaders adapted the intervention to the needs of the group:

*The times were relaxed as far as the welcoming was concerned (…) however (…) the singing leader's programme was very structured, so there was a common thread. (Mental health professional Group 2)*

Tools such as scarves, wrapping paper and maracas were used to interact with and calm the babies. In one group, where their use was not planned, mothers made maracas following the singing leader’s instructions:

*Singing only was a bit challenging (…) we made small maracas (…) Then every now and then we did some little activities, which I had not planned, to involve them even more also in the relationship with the babies. (Singing leader Group 3)*

#### 3.1.5 Implementation process

##### 3.1.5.1 Strategies adopted

The offer of the singing intervention was well received. On enrolment, mothers were given a clear explanation of what the intervention entailed, that no singing or musical skills were required, and where the classes would take place. The subject of PPD was carefully introduced. The women were reassured that the singing group was aimed at new mothers with similar emotional experiences and their babies:

*It was a little difficult at first, in the sense that you read about PPD and it’s scary (…). Because the woman doesn’t accept it right away. (Referral Group 3)*

The professionals valued the clear definition of roles and tasks in delivering the intervention, the continuity provided by the presence of the same professionals at each session, and the appropriate venue:

*The venue, from all points of view (…) they come and don’t have to think about anything else. Are they thirsty? We have water (…) there were those who came by bus, those who came by metro, those who came by car, and in any case (…) you can find a parking. And the fact that (…) they always met the same faces. (Support staff Group 2)*

The implementation within the Family Care Centres was strategic because the multi-professional team had the skills and training to identify, address and take care of women with PPD symptoms. Moreover, the study provided an opportunity to strengthen the integrated and multidisciplinary work at Family Care Centres:

*There was a real integration between different professional figures who carried out this work together, but harmoniously. (Support staff Group 3)*

The intervention was also an opportunity for enrichment and professional satisfaction for the staff members involved:

*It was very, very enriching. Very interesting. (Support staff Group 3)*

*It was a great satisfaction and also a confirmation that we were doing something good to help mothers in the postnatal period. We felt that a lot. (Project manager Group 1)*

According to one local project manager, “Music and Motherhood” could be an opportunity not only to promote the empowerment of the participating mothers, but also to establish a more structured peer support among new mothers and collaboration between Family Care Centres and third sector:

*In the future this could be something that is offered on a large scale to the mothers, which could then stimulate the mothers themselves to also become (…) support for others, and the other aspect is that the support of the third sector is indispensable. (Project manager Group 2)*

The central coordination by Istituto Superiore di Sanità facilitated implementation by monitoring the whole process, scheduling activities, and being constantly in contact with the local teams:

*The work that you [Istituto Superiore di Sanità] have done, it’s not that it’s taken for granted (…) the whole part of how this group is created, in what relationship with the territory and the already existing structures. (Singing leader Group 3)*

##### 3.1.5.2 Training

The training was evaluated as “very, very, very interesting” (Singing leader, Group 1) and relevant. The duration was appropriate and the content was clear. Moreover: “[it was] very useful to have an overview in terms of methods, objectives, resources (…) for my role, it was extremely useful to do this training” (Link worker). Some local professionals suggested that a training provided in the local language by professionals who have implemented the intervention in the local context could facilitate scaling it up:

*In a future perspective the testimony of the locals will be further helpful to those who decide to implement it. (Project manager Group 1)*

Professional singing leaders would have appreciated receiving “videos of their sessions and more examples of what they sing. Maybe also a sharing of material” (Group 1), “a small collection [of music used] (…) the lyrics, the chords” (Group 3), or even more examples of the suggested song types, such as “round songs” or “action songs” (Group 2).

#### 3.1.6 Costs and sustainability

##### 3.1.6.1 Associated costs

Regarding Family Care Centres, the main burden of implementation was the time availability of the health professionals, as “dedicated staff is needed” (Project manager, Group 2). The commitment for each class “was about 2 h” (Support staff Group 3); it was necessary to have one professional within the Family Care Centre to assist the professional singing leader and another to help with reception. In two Family Care Centres, all the health professionals contributing to the intervention were already on staff at the Local Health Unit, while one group employed an additional midwife. In line with safeguarding procedures, a mental health professional from the Local Health Unit was involved in monitoring the mothers. Although this may be a barrier to implementation in health services burdened with staff shortages, it was noted that it may reduce burden in other ways:

*A standard medical-psychological intervention costs a lot more … So anyway it reduces costs and de-medicalises an area that is so much in need of de-medicalisation. (Project manager Group 1)*

Human resources costs also include the professional singing leader and, where needed, a link worker. A start-kit with small musical instruments, mats, socks, scarves, and refreshments is also recommended.

For mothers, the cost of participation was travel to the venue or occasional babysitting for older children. Some participants who were no longer on maternity leave were given paid leave to attend the final sessions.

##### 3.1.6.2 Mothers’ intention to adopt the intervention

At the end of the intervention, mothers continued or expressed willingness to continue practising singing. Some of them kept in touch and even met up to sing together:

*I continue to sing with the baby (…) I think it’s an opportunity for other mum for continuity (…) yesterday [I was at] my house with 5-7 mums. I sang, it was nice, then, that music (…) and all the mums, I talked, you have to be together. And if you have a continuation, it’s so good and beautiful. (Mother Group 2 ID 8)*

Each group spontaneously created a WhatsApp group. Mothers used the learnt songs when the children were most upset, and to manage moments of emotional distress:

*Now we sing a lot after this experience (…) it helps a lot not to think about bad things or to overcome fears, uncertainties (…) if we sing one of the songs we learnt during the course (…) [the baby] calms down, smiles. (Mother Group 3 ID 14)*

Interest was also expressed in finding “other projects and services in the area that may be similar” (Mother Group 1 ID 4).

##### 3.1.6.3 Long-term sustainability

The intention to replicate the “Music and Motherhood” experience and support its continuation came from both the health professionals and the professional singing leaders: “seeing the results motivates you, gives you satisfaction (…) yes, I would like [continue]. Definitely” (Singing leader Group 2). It was also expressed that it would be a shame for this intervention to be an isolated experience, and that work should begin to explore how to run another group:

*What do we do? Immediately, we start again with another group… it has to be repeated (…) it has to be implemented, it has to be disseminated. (Project manager Group 1)*

The Family Care Centre proved to be a suitable context and the intervention seems to be reproducible within the system in which it was implemented in the context of this study. Due to their multi-professional nature, Family Care Centres made “Music and Motherhood” sustainable with the available human resources and without dedicated funding. The transition to a stable offer requires taking into account the time load of the staff involved: “(…) to realise it as a service, the problem is resources, personnel” (Project Manager Group 2).

##### 3.1.6.4 Contextual or structural factors

The implementation was possible within the project timeframe because routine procedures at the level of the Family Care Centre include screening for PPD:

*The involvement of the Local Health Unit is crucial because you have a facility that can identify in a timely manner the characteristics required by the project. (Link worker)*

The context of COVID-19 partially influenced the delivery of the intervention. In fact, the choice of venue fell on spaces not dedicated to patients, even near to the Family Care Centre in two cases out of three. This potential barrier made it possible to think carefully about venue identification, and to find viable solutions. The suitability of the venue is a factor facilitating successful implementation and participation. The choice of the public music library in Turin, in particular, was a facilitating factor: “we were favoured by this beautiful environment we were in” (Singing leader).

It was suggested that research participation represented a barrier for some: “the experimental design of the study seems to have inhibited some of these women” (Project manager Group 1). Data protection restrictions prevented the sharing of images or videos; it was suggested that future initiatives should allow mothers to keep memories of this group experience: “the mothers would have liked to have some memories, even audio-visual ones” (Singing leader Group 1).

### 3.2 Quantitative component

The median number of sessions attended by the 23 participant mothers was nine (mean 8.7, SD = 1.1) out of the total 10. The 10-week intervention was completed by all 23 mothers.

**Figure 2:**
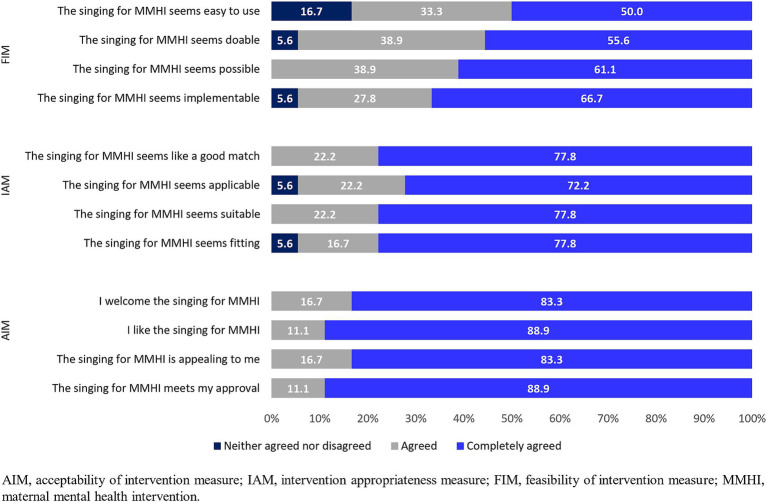
Proportion of respondents who neither agreed nor disagreed/agreed/completely agreed with survey questions about the acceptability (AIM), appropriateness (IAM), and feasibility (FIM) of the “Music and Motherhood” intervention.

Figure 2 reports the proportions of respondents who neither agreed nor disagreed, agreed, completely agreed with the implementation measures. Measure of intervention acceptability obtained the highest scores [median (IQR): 20 (20-20)] with almost 90% of professionals completely agreed to like and approve the intervention.

The median value (IQR) of measure of intervention appropriateness was 20 (20-22), with about three-quarters of respondents completely agreeing with all 4 items of the appropriateness measure IAM.

The feasibility of intervention measure had the lowest score (median (IQR): 19 (16-20) with half of professionals completely agreeing that the intervention was easy to use.

## 4 Discussion

This is the first study providing insights into the implementation of an evidence-based arts and health intervention in the Italian NHS.

A seminal systematic review by Dennis and Chung-Lee identified women’s difficulty to disclose their negative feelings, fear of stigma and reluctance to use mental health services as the main help-seeking barriers for PPD, while maternal treatment preferences included counselling from non-judgmental, unhurried health professionals and support from other women with children (5). Overall, the “Music and Motherhood” intervention implemented in Italy has shown promise in overcoming barriers to care. It was well accepted by new mothers with symptoms of PPD, professionals from the community primary care services, and the professional singing leaders involved. A conceptual framework of six overarching themes was identified to describe the milestones of project implementation. In terms of acceptability, women’s trust in the referring health professionals was central to their decision to participate in the singing groups, which proved to be an inclusive proposal for women from different socio-cultural and linguistic backgrounds. The appropriateness of “Music and Motherhood” is the result of several elements: (i) the group setting, which gives women the opportunity to be supported by peers; (ii) the singing, which offers new mothers a de-medicalised approach to expressing their emotions and a channel for communicating with their child; (iii) the integration of the singing intervention within the health service, which allows for a holistic response to the needs of women experiencing depressive symptoms in the postnatal period. Weekly attendance at the singing classes in well-equipped and easily accessible venue proved to be feasible. In terms of fidelity, the implementation reflected the adaptation to the Italian context, which included the participation of a health professional within the Family Care Centre in the classes and flexibility in the timing. In recruiting participants, the referrers’ ability to identify women with postnatal emotional distress and offer the intervention as a valuable opportunity was instrumental in encouraging participation. The health professionals’ understanding of the needs of new mothers enabled them to quickly identify and set up suitable venues, while their experience of working in a multi-professional context made it easy to work with the singing leaders, taking advantage of different roles and perspectives. The provision of appropriate training enabled the local teams to feel ready to deliver the innovative intervention, while the central implementation team enabled them to seek advice when needed and to focus on implementation without being overburdened with research tasks. The high average attendance of mothers in the singing classes, similar to that of mothers in the UK trial (7.2 sessions out of 10) (13), the lack of drop-outs, and the positive experiences reported by the women and staff involved suggest that the proposed intervention met the needs of its target population and should be made more widely available.

Compared with the adaptation carried out in Denmark and Romania, the Italian experience involved the health services more directly and in the whole process. This made it possible to take advantage of the previous collaboration between the coordinating research institute and the participating health services, as well as the care relationship between the target population and the health professionals involved, which was reflected in the speed of the referral process and overall implementation. Linguistic translation of the intervention was not a barrier to the implementation of the intervention in Italy, in line with the findings in the other two countries.

According to the WHO stepped care model (20), which focuses on maternal and child health services as a unique opportunity to offer mental health support to women during the perinatal period, Family Care Centres are the women’s health services that, if adequately supported, could play a significant role in the promotion of perinatal mental health and in the management of PPD with mild to moderate symptoms in Italy (31). The “Music and Motherhood” intervention that addresses women with PPD symptoms who are in contact with these services therefore seems particularly promising. Further implementation and evaluation of the group singing intervention for PPD could draw on the experience of those countries that already use non-clinical community resources for the health and well-being of patients within cost-effective social prescription pathways (32).

The willingness to continue singing classes was unanimous. However, the sustainability of the intervention over time would require additional resources. As it was considered essential that mothers participate free of charge, the cost to the health service would include the time of health professionals to support the intervention, the payment of the professional singing leaders and the provision of the intervention tools. The lack of resources in the Family Care Centres and the lack of a training package in the local language were identified as the main barriers to scaling up “Music and Motherhood” in Italy. The quantitative findings confirmed the qualitative ones. The high scores on the acceptability and feasibility measures confirmed the professional’s willingness to implement the intervention, while the lower score recorded for the feasibility dimension may reflect the lack of resources described above.

Our results must be interpreted in the light of some limitations. Firstly, our description does not include the contribution of international coordination and exchange with the other countries involved, which is however reported elsewhere (23). Another limitation is the lack of a detailed cost assessment of the implementation of “Music and Motherhood” compared to other treatment options. The data collected will hopefully allow a better analysis of this aspect in the future. The limited data on the socio-economic conditions of the participating mothers, and the limited number of women involved in the qualitative assessment of participation feasibility represent a limitation to the generalisability of the results. Future implementation of the intervention could address this shortcoming. Finally, the Family Care Centres involved in the implementation are not representative of the national network of these services, but rather of those that have included perinatal mental health promotion and support among their main activities.

Our study adds to the evidence that an arts and health-based intervention proven effective in one context can be successfully adapted to another culture and language. Successful integration in a primary care setting requires a well-defined protocol, training and support for the professionals involved, facilitated by a central implementation team. Professionals and participant mothers agreed on several key elements of their experience: attention to mothers and their needs, the ability to create a welcoming environment, and the availability of a non-judgmental and empathic setting. The identification of specific health needs to which the artistic intervention can respond, the collaboration of health professionals and artists in the implementation process, and adequate funding are key steps in the transition from the project to the programme dimension. Within global arts and health policy and implementation, there are challenges to constructing the intersectoral infrastructure across settings needed to successfully implement arts and health interventions, particularly in relation to gaining buy-in from the health sector due to the pressures faced by healthcare professionals. In addition to the talent and abilities of the professional singing leaders, our study uniquely engaged a high-level of commitment, time, and resources from healthcare services and staff, all of which were crucial to its success. This suggests that future research and policy in arts and health needs to further explore how to strengthen collaborations across the arts and health sectors in order to enable the scale up of this effective intervention into other cultural settings and supporting more women globally.

## Data Availability

The datasets in this article are not publicly available to preserve the anonymity of participants. Requests to access the datasets should be directed to ILe, ilaria.lega@iss.it.

## Footnotes

^1^Jameel Arts & Health Lab. Music and Motherhood. (2024). https://www.jameelartshealthlab.org/research/research-projects/music-and-motherhood-ucl [Accessed May 28, 2024].

## Group members of the Italy and GLOBUS Music and Motherhood Groups

The Italy Music and Motherhood Group

Central Implementation Team

Ilaria Lega, Ilaria Luzi, Claudia Ferraro, Simona Mastroeni, Silvia Andreozzi, Serena Donati, Istituto Superiore di Sanità, Rome, Italy.

Local Implementation Team, Este

Pietro Grussu, Emanuela Tezzon, Stefania Brun, Azienda ULSS 6 Euganea, Este, Italy.

Valentina Cavazzana, Dandelion Associazione di promozione sociale, Maserà di Padova, Italy.

Local Implementation Team, Rome

Patrizia Proietti, Patrizia Magliocchetti, Emanuela Menichelli, Erika Natale, Alessandra Maria Monti, Daniela Aiello, Patrizia Sallese, Cinzia Di Giuseppe, ASL Roma 2, Rome, Italy.

Chiara Monaldi, Studio Lago - Il centro della voce, Rome, Italy.

Local Implementation Team, Turin

Cristina Biglia, Giulia Maurino, Daniela Morero, Silvio Saccani, Maria Crisetti, Giulia Mortara, Giuseppina Viola, Maria Clara Zanotto, ASL Città di Torino, Turin, Italy.

Catterina Seia, Paola Matossi, Rossana Oreggia, Cultural Welfare Center, Turin, Italy.

The GLOBUS Music and Motherhood Group

Project Coordinators

Calum Smith, Nuffield Department of Population Health, University of Oxford, Oxford, United Kingdom/ Consultant, Behavioural and Cultural Insights Unit, World Health Organization Regional Office for Europe, Copenhagen, Denmark.

Katey Warran, Social Biobehavioural Research Group, Research Department of Behavioural Science and Health, University College London, London, United Kingdom/ School of Health in Social Science, University of Edinburgh, Edinburgh, United Kingdom.

Nils Fietje, Behavioural and Cultural Insights Unit, World Health Organization Regional Office for Europe, Copenhagen, Denmark.

Training Coordinator

Lorna Greenwood, Breathe Arts Health Research, London, United Kingdom.

National Project Team Members

Hanna Ugron, Rarita Zbranca, Centrul Cultural Clujean, Cluj-Napoca, Romania.

Louise Frøkjær Carstens, Den Kreative Skole, Region Midtjylland (Central Denmark Region), Viborg, Denmark.

Mikkel Ottow, Region Midtjylland (Central Denmark Region), Viborg, Denmark.
