# Supplementary material for: Implementing a group singing intervention for postpartum depression within the Italian health service
Source: Front Med (Lausanne). 2024 Oct 21;11:1461965. doi: 10.3389/fmed.2024.1461965 (PMC11532103; doi:10.3389/fmed.2024.1461965)
Supplement: Supplementary file 1 [file Table_1.DOCX]

Supplementary Material

Implementing a group singing intervention for postpartum depression within the Italian health service

Ilaria Lega^1*^, Ilaria Luzi^2^, Simona Mastroeni^1^, Claudia Ferraro^1^, Silvia Andreozzi^1^, Serena Donati^1^, Pietro Grussu^3^, Valentina Cavazzana^4^, Patrizia Proietti^5^, Patrizia Magliocchetti^5^, Chiara Monaldi^6^, Cristina Biglia^7^, Rossana Oreggia^8^, Catterina Seia^8^, Calum Smith^9,10^, Katey Warran^11,12^, Nils Fietje^13^ and the Italy° and GLOBUS^ Music and Motherhood Groups

*** Correspondence:** Corresponding Author: ilaria.lega@iss.it

# Supplementary Table

**Example of quotes for themes/subthemes**

| **Theme/Subtheme** | **Example quote** |
| --- | --- |
| **Acceptability** |  |
| Reasons for taking part | *To give myself a different possibility and to find other mothers who were experiencing my situation (...) The fact of being together (…) to be able to share, without feeling judged in my feelings. (Mother Group 1 ID 4)* |
| Content and structure of the intervention | *I was [impressed], in short, to see this group grow. Because at the beginning, they were a bit sceptical, they were quite closed... but as they did these singing classes and there was an important growth in terms of the cohesion of the group (...) which was very diverse and included participants with challenging personal experiences. (Support staff Group 3)* |
| **Appropriateness** |  |
| Relevance to PPD symptoms management | *There are things not detectable by the instruments we used for the evaluation, [things] which we touched with our own hands: that is, seeing them greet each other, relate to each other at the end of the last session (Project manager Group 1)* |
| Core programme contents | *An opportunity to get together, where one of the biggest problems after birth is to leave the house. And to share the same experience with peers. Therefore, the benefits are multiple, going beyond the singing, which is also therapeutic. To meet regularly, to have a commitment, to comply with a commitment made in a consistent manner (...) I have nothing but words of praise for this kind of intervention. (Project manager Group 1)* |
| **Feasibility** |  |
| Attendance | *Let’s say for us, who often do activities with new mothers, we adopt this once a week frequency because we have seen that it is neither too little nor too much (…) These mothers have never said to us:* Why didn't you do it twice a week? *Or, on the other hand, that once is too much. We’ve understood that once a week is good. (Support staff Group 1)* |
| **Fidelity** |  |
| Receipt and adaptations | *In general, what I thought I was doing from the beginning was more or less always done. (...) That is, apart from the requests that were made to me. When they asked me* Will we sing in Italian? Will we sing [title of the song]? *(…) And we did what they asked. (Singing leader Group 2)* |
| **Implementation process** |  |
| Strategies adopted | *One person to take care of the logistical part, so that everyone has their own clearly defined role, and when [the staff] introduce themselves to the mothers, they know who does what (...). The mothers need continuity, and finding the same faces created this activation of trust and confidence because the people were the same, and there was also continuity of dialogue (...) Continuity is a key element. And then (...) relieving the mothers of any organisational burden. (Link worker)* |
| **Costs and sustainability** |  |
| Associated costs | *A midwife is needed. (Link worker)*  *To set up the room, we got the material quite quickly. The budget is needed to set up the buffet for each weekly class. (Support staff Group 3)*  *The cost of the instruments must be taken into account. (Singing leader Group 3)* |
| Mothers’ intention to adopt the intervention | *They however created a relationship between them, yes, they created their WhatsApp group, to find each other outside, to inform each other of other initiatives (...) that’s definitely a positive thing. (Singing leader Group 1)*  *It was so engaging the way we learned these songs, that by now, I mean, my husband who wasn't there knows them, and even my husband plays them on the guitar, now at home. So, it really became a part of us (…) And relationships continue with some of the participants (…), also very, very close friendships. So yes, let’s say I brought home a lot of baggage. (Mother Group 1 ID 8).* |
| Long-term sustainability | *This is really a work that starts in the community. And this is fundamental because this prevention done for these mothers (...) will avoid a series of subsequent specialised courses, a series of future consultations for their children (…). The return would be enormous. (Project manager Group 2)* |
| Contextual or structural factors | *The midwifery colleagues have shown themselves to be available and sensitive. We are lucky to have already been trained and sensitised in previous years [by introducing] (…) the screening for perinatal depression (...). This gave us the extra motivation to measure ourselves in this project as well. (Project manager Group 3)*  *If such an offer is made in a proactive way in the FCCs, I think it would be an added value (...) in my opinion. For the way we are organised in Italy, the FCCs could be a very good place for a broad adherence, removing all the stigmatisation that there is (...) and therefore also the label. The way (…) services are organised in Italy (...), a community service like the FCC is just made for it. (Project manager Group 1)* |

**
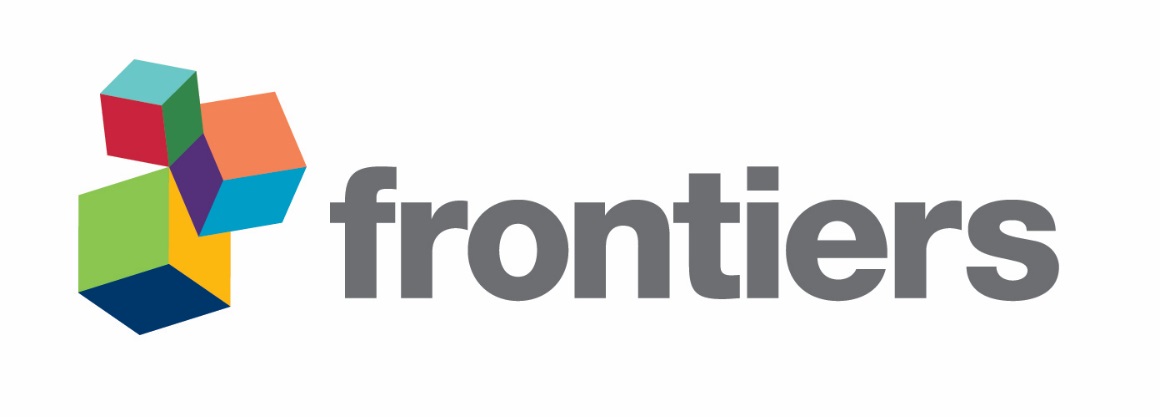
**
